# Hypoxia Inducible Factor-1α: The Curator of Gut Homeostasis

**DOI:** 10.3389/fcimb.2020.00227

**Published:** 2020-05-15

**Authors:** Tarun Kumar, Rajesh Pandey, Nar Singh Chauhan

**Affiliations:** ^1^Department of Biochemistry, Maharshi Dayanand University, Rohtak, India; ^2^Genomics and Molecular Medicine, CSIR-Institute of Genomics and Integrative Biology (CSIR-IGIB), New Delhi, India

**Keywords:** HIF-1α, host microbial crosstalk, gut health, gut epithelial integrity, human gut microbiome, regulation of gene expression, SCFA

## Abstract

The human gut microbiome is a stratified and resilient ecosystem co-inhabited by a diverse and dynamic pool of microorganisms. Microbial selection, establishment, and colonization are modulated through a complex molecular network of host-microbial interactions. These molecular bioprocesses ensure the taxonomic composition of the mature human gut microbiome. The human gut microbiome plays a vital role in host health; otherwise, any microbial dysbiosis could predispose to the onset of physiological and metabolic disorder/s. Focussed research are being carried out to identify key molecular agents defining gut homeostasis. These molecules hold the potential to develop effective therapeutic solutions for microbial dysbiosis-associated human disorders. Of these, Hypoxia-inducible factor-1α (HIF-1α) is a central player in host-microbial crosstalk to maintain gut homeostasis. Human gut microbial metabolites regulate its cellular stability, which in turn regulates various cellular processes required for the stable gut microbiome. In the present review, an effort has been made to summarize the key role of HIF-1α to maintain gut homeostasis.

**Highlights:**

- Explain the molecular process of host microbial molecular interactions.
- Establish the explicit role of HIF-1α in intestinal epithelial integrity and gut health.
- Regulation of HIF-1α by human gut commensals and vice a versa.
- Regulation of the host immune response for survival and colonization of human gut commensal.

## Introduction

The human body is a permanent yet dynamic address to a variety of microorganisms living within and outside the human body (Sender et al., 2016). All these microresidents have a commensal relationship with the host and constitute the human microbiome (Laterza et al., 2016). The diversity of microbes varies with host anatomical locations wherein the human gut microbiome is a highly diverse and well-studied system (Thursby and Juge, 2017). In general, the human gut microbiome is predominantly occupied by *Firmicutes* and *Bacteroidetes* followed by *Proteobacteria, Actinobacteria*, and *Acidobacteria* that show inter-individual variation. In addition, microbes show intra-individual variability but the human microbiome is considered resilient in nature as well (Lozupone et al., 2012). Colonization of microbes starts with birth that progressively gets more diverse and stable with the host growth (Rogier et al., 2014). The maturation of the human gut microbiome is based on complex and dynamic host-microbial interactions being influenced by genetic (Morán-Ramos et al., 2019) and epigenetic factors (Miro-Blanch and Yanes, 2019). Despite a complex nature, their interactions provide many health benefits that lead to better survival of the host (Nicholson et al., 2012). These interactions modulate various metabolic and physiological processes (Yadav et al., 2018). Though the human gut microbiome is resilient, various factors could influence human gut microbial dysbiosis (de la Cuesta-Zuluaga et al., 2019), leading to the onset of various physiological and metabolic disorders (Petersen and Round, 2014). Despite a plethora of information about the human gut microbiome, composition, significance in host physiology, association with onset and prevention of diseases, we are progressing toward understanding host-microbial interactions concerning microbiome homeostasis and dysbiosis. Several key questions like,

- how the host differentiates among commensals and pathogens,
- what are the factors that enable the host to maintain healthy microbiome homeostasis,
- what are the molecular changes that disrupt healthy microbiome homeostasis during microbial dysbiosis; are still unanswered.

These critical questions could be helpful to develop sustainable therapeutic solutions for microbial dysbiosis-associated human disorders. Answer to these key questions could be obtained by revisiting molecular studies defining human gut microbial recognition by host immune system, host immune system maturation by the human microbiome and protective strategies employed by human gut microbes toward commensalism. Several molecular components were identified in this regard with Hypoxia-inducible factor-1α (HIF-1α) being one of the core player in maintaining gut microbiome homeostasis. In the current review, we have made an effort to summarize the explicit role of HIF-1α in gut microbiome maintenance.

## Regulation OF HIF-1α Function

**Figure 1:**
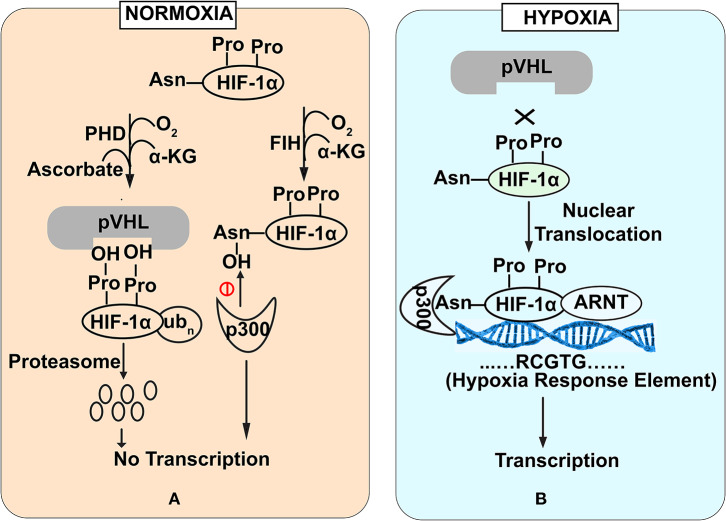
Stability of HIF-1α subunit under Normoxia **(A)** and Hypoxia **(B)** conditions. Under normoxia **(A)**, prolyl hydroxylase domain enzymes mediate HIF-1α subunit degradation of HIF by hydroxylation-induced proteasome—mediated pathway. In contrast under hypoxia **(B)**, molecular oxygen is not available for hydroxylation that favors HIF-1α stabilization. Stable HIF-1α binds with HIF-1β subunit to bind with core promoter sequence of HIF-1α target genes.

HIF-1α is a regulator of oxygen homeostasis in metazoans (Semenza, 2012). Structurally, this transcription factor is a dimer of oxygen responsive -α subunit and a constitutively expressed -β subunit (or aryl hydrocarbon receptor nuclear translocator (ARNT) (Lee et al., 2004). The regulation of HIF-1α is mediated through the stabilization and destabilization of α-subunit that is governed by the oxygen concentration in intestinal lumen. In physiological terms, 10–21% oxygen tension is defined as normoxia while 1–5% oxygen tension is defined as cellular hypoxia. Hypoxia could be induced by various cellular bioprocesses like aerobic respiration (Kelly et al., 2015), respiratory burst by immune cells (Glover and Colgan, 2011). During normoxic conditions, prolyl hydroxylase domain enzymes (PHDs) mediate -α subunit degradation by hydroxylation-induced proteasome-mediated pathway. PHDs are dioxygenases which require ascorbate and iron as cofactors (Durán et al., 2012). They exploit molecular oxygen to hydroxylate, one of the two proline residues on the HIF-1α subunit in an α-ketoglutarate dependent manner (Taniguchi et al., 2014). As a part of the process, one oxygen atom from the molecule is incorporated into prolyl residue, while the incorporation of another oxygen atom into the α-ketoglutarate leading to it's splitting into succinate and CO_2_ (Kaelin and Ratcliffe, 2008). Hydroxylated HIF-1α subunit acts as a recognition motif for the von Hippel-Lindau (pVHL) tumor suppressor protein, leading to E3 ubiquitin ligase complex recruitment followed by its proteasomal degradation (Kaelin and Ratcliffe, 2008) (Figure 1A). In contrast, under hypoxic conditions, molecular oxygen is not available for hydroxylation. Therefore, a low oxygen environment does not favor HIF-1α subunit hydroxylation, resulting in its stabilization. This stable HIF-1α subunit in a low oxygen environment binds to the HIF-1β subunit to form a heterodimer. This hetero dimer binds at the core sequence RCGTG of HIF-1α response elements (HREs) of HIF-1α target genes promoter (Mole et al., 2009) (Figure 1B). Although, in a contrasting scenario, HIF-1α stabilization in immune cells also occurs during normoxic conditions. Induction of macrophages with bacterial lipopolysaccharide (LPS) or Tumor necrosis factor-α (TNF-α) has been found to promote the accumulation of HIF-1α protein in macrophages (Palazon et al., 2014). Further, gut commensals could lead to the stabilization of the HIF-1α in the gastrointestinal tract. *Firmicutes, Bacteroidetes*, and *Clostridial* cluster in the human gut convert dietary fibers and resistant starch into short-chain fatty acid (SCFA) (Parada Venegas et al., 2019). SCFA enters the nucleus of colonocytes and acts as a histone deacetylase inhibitor. SCFAs stimulate pyruvate dehydrogenase kinase (PDK) that in turn inactivates the pyruvate dehydrogenase complex. Inactivated pyruvate dehydrogenase complex cannot convert pyruvate to acetyl-CoA to meet cellular energy requirements. Thus, colonocytes start generating acetyl-CoA through β-oxidation of the SCFA. Increased oxidative respiration induces physiologic hypoxia which in turn tends to stabilize HIF-1α (Kelly et al., 2015). In addition to the SCFA mediation HIF1α stabilization, human gut commensals also regulates the PHDs activity to stabilize HIF1α. PHDs require iron for their catalytic role to destabilize HIF1α and absence of iron affects PHDs activity. Human gut commensals could secrete siderophores to quench iron from intestinal lumens making it unavailable for PHDs and allowing HIF1α stabilization (Golonka et al., 2019).

## Functional Role Of HIF- 1α

**Figure 2:**
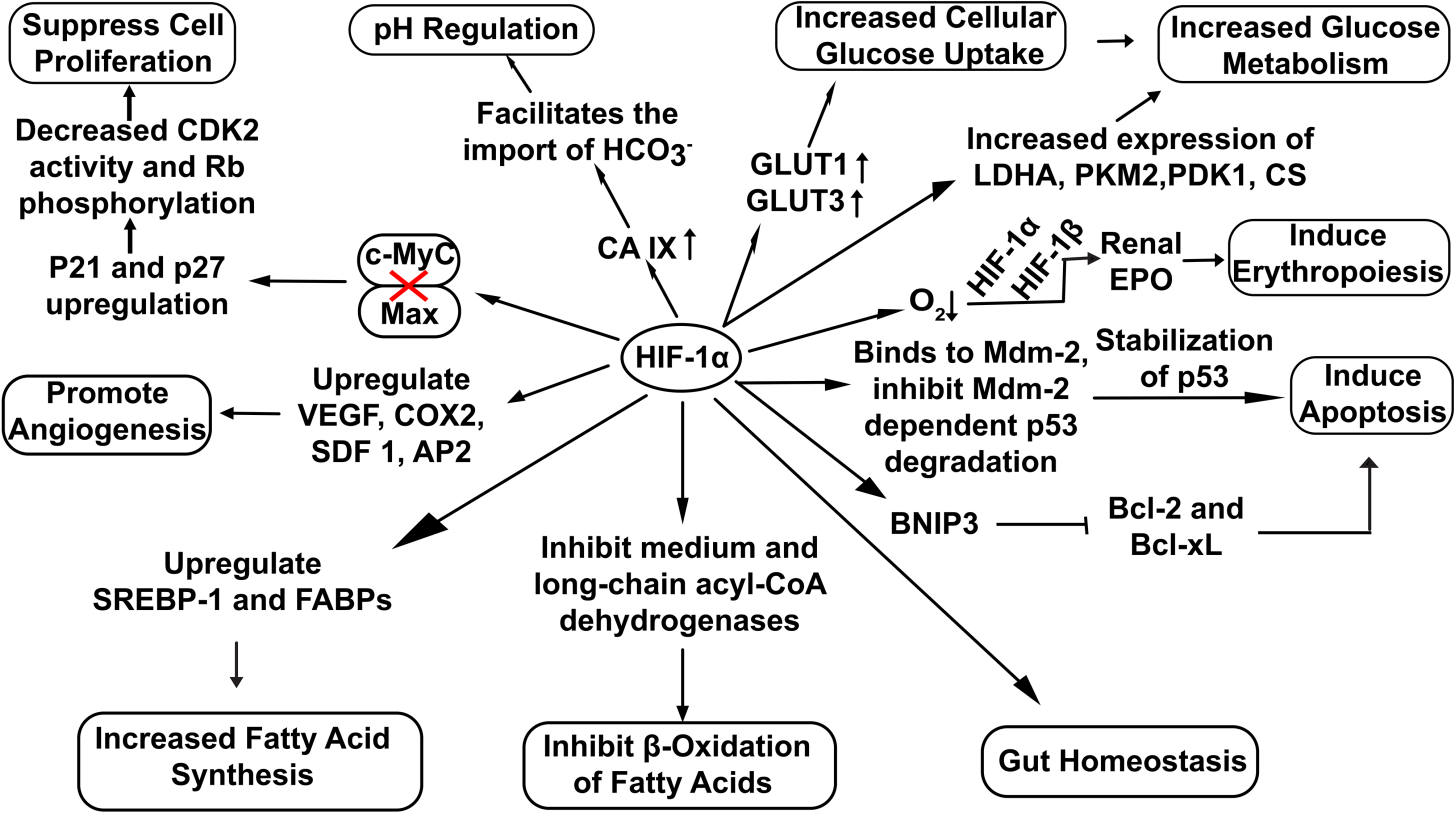
Overview of cellular functions regulated by HIF-1α. Stable HIF−1α regulates the expression of various genes involved in diverse cellular bioprocesses. (Here: EPO, Erythropoietin; CDK2, Cyclin-Dependent Kinase 2; Rb, Retinoblastoma protein; VEGF, vascular endothelial growth factor; COX2, Cyclooxygenase 2; SDF 1, Stromal Derived Factor 1; SREBP-1, Sterol Regulatory-Element Binding Protein-1; FABPs, Fatty Acid Binding Proteins; AP2,Angiopoietin 2; LDHA, Lactate Dehydrogenase A; PDK1, Pyruvate Dehydrogenase Kinase 1; CS, Citrate Synthase; PKM2, Pyruvate Kinase M2; BNIP3, BCL2/adenovirus E1B 19 kDa interacting protein 3; Bcl-2, B-cell lymphoma 2; Bcl-xL, B-cell lymphoma-extra-large).

HIF-1α has been extensively involved in a broad range of cellular functions like pH regulation (Sedlakova et al., 2014), increased glucose uptake (He et al., 2014), erythropoiesis induction (Haase, 2013) and lipid metabolism (Zhang, 2015) (Figure 2). Recent studies also highlight the potential role of HIF-1α in the maintenance of gut homeostasis. It not only regulates the integrity of the gut epithelial barrier but also leads to better survival of gut microresidents (Fan et al., 2015).

### Maintains Epithelial Barrier Integrity

The intestinal epithelium is the primary site of host-microbe interactions modulating physiological outcome. To prevent microbial invasion and to protect microbe from the host immune system, the barrier function needs to be tightly regulated. The human gut, in general, provides favorable conditions for stabilization of HIF-1α to maintain gut homeostasis. The stabilization of HIF-1α affects diverse functions within the human gut.

#### Epithelial Barrier Regulation by HIF-1α

**Figure 3:**
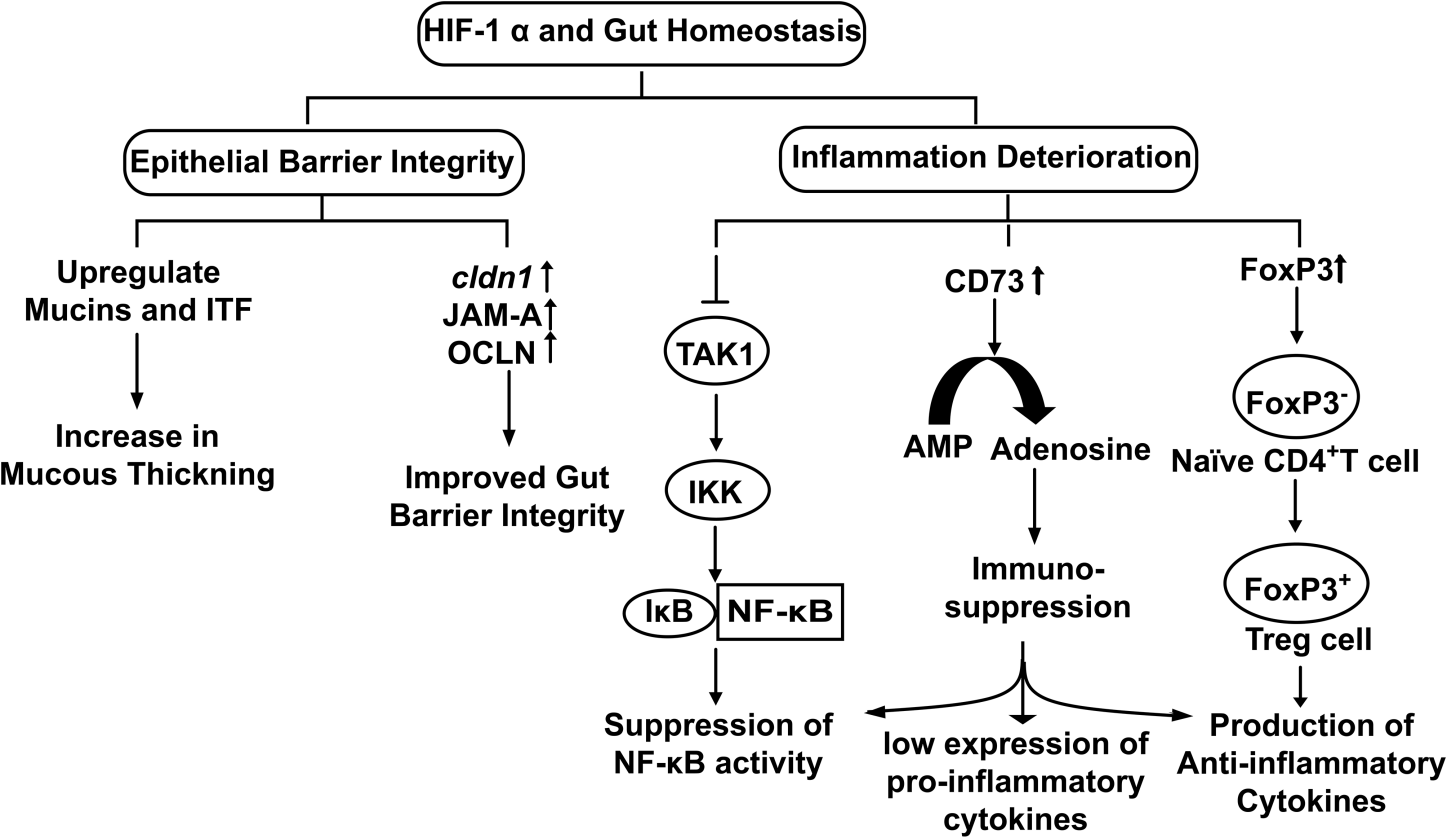
The physiological role of HIF-1α in the maintenance of Gut Homeostasis. Hypoxia stabilized HIF-1α up regulates the expression of genes involved in epithelial integrity and down regulates cellular inflammation.

Stabilization of HIF-1α upregulates the transcription of Claudin-1 (*CLDN1*), a tight junction membrane protein maintaining tight junction structure and functions (Van Itallie and Anderson, 2014) (Figure 3). Enhancement of gut barrier function by hypoxia was confirmed by growing T84 cells in a hypoxic environment. An increase in transepithelial electrical resistance (TEER) along with the decrease in diffusion of fluorescein isothiocyanate (FITC) labeled dextran was observed in the cells grown under hypoxic conditions (Muenchau et al., 2019). Additionally, junctional adhesion molecule-A (JAM-A) and occludin (OCLN) were also found upregulated under hypoxic conditions. Such examples of increased barrier functions were found to be HIF-1α-dependent, highlighting the direct role of HIF-1α in maintaining the integrity of intestinal epithelium (Muenchau et al., 2019). Increased barrier integrity could be involved in immune-suppressive effect as improved gut barrier seals the paracellular pathway and inhibit the activation of underlying immune cells which otherwise could result in conditions of colitis (Chelakkot et al., 2018). Additionally, Dimethyloxalylglycine (DMOG), an inhibitor of PHDs and Factor Inhibiting HIF (FIH) have been shown to ameliorate dextran-sodium sulfate (DSS)-induced colitis by stabilizing HIF-1α (Cummins et al., 2008). On the other side of physiological role of gut integrity, in the case of IBD, increased barrier permeability results in inflammation and altered microbial composition (Martini et al., 2017). Butyrate in Caco-2 cells was found to enhance O_2_ consumption and stabilize HIF-1α which in turn reduced barrier permeability (Kelly et al., 2015). Similarly, butyrate was found to upregulate tight junction proteins in HIF-1α dependent manner to improve barrier integrity, reducing inflammation by inhibiting microbial translocation in mice (Fachi et al., 2019). Taking the findings together, it highlights that HIF-1α up-regulates tight junction proteins to enhance the epithelial barrier integrity to reduce gut inflammation and facilitate microbial colonization.

#### HIF-1α Induced Mucins Facilitate Colonization of Commensals

The mucous layer consists of a dense inner layer that restricts the bacterial attachment and a loose outer layer, residence of gut commensals (Johansson et al., 2008). Intestinal mucus is mainly composed of glycoproteins that are specifically O-glycosylated called mucins (MUC) having a common PTS domain rich in proline, threonine, and serine. The stomach mucous layer mainly consists of MUC1, MUC5AC, and MUC6 while MUC2 is a major component of the mucous layer of the small intestine and colon (Holmén Larsson et al., 2013). The carbohydrate-rich mucous layer acts as a growth substrate for the resident microbes. Further, commensals can induce MUC2 production to increase the thickness of the mucous layer that not only provides a space to gut colonizers but also provides pathogen resistance to the host. Probiotic bacteria such as *Lactobacillus* can stimulate mucin production (Caballero-Franco et al., 2007). *B. thetaiotomicron* and *faecalibacterium prausnitzi* were also found to accelerate goblet cell differentiation (Wrzosek et al., 2013).

The mucous layer secreted by goblet cells in the gut is an essential part of the innate defense system. It forms a physical barrier to restrict direct access of pathogens to the gut epithelium (Johansson et al., 2008). Mucins interact with trefoil factors to enhance the mucosal barrier (Aamann et al., 2014). It is also well-characterized that human gut commensals release SCFA that in turn stabilizes HIF-1α by inducing metabolic hypoxia in colonocytes. Stabilized HIF-1α upregulates the expression of MUC2 (Dilly et al., 2016), MUC3 (Louis et al., 2006) and intestinal trefoil factor (ITF) (Glover et al., 2016) (Figure 3). Furthermore, it has been observed that mice lacking HIF-1α showed less organized and diffused mucin granules, suggesting HIF-1α expression is required for mucin processing (Whitney et al., 2016).

#### Inflammation Deterioration

The immune system plays a vital role in gut homeostasis. Exposures to microbes have been shown to activate host immune system, whilst a hyper-activate immune system could result in detrimental effects to the host itself. HIF-1α has been explored for its immune suppressive potential and as a mediator of gut harmony.

#### NF-κB and HIF-1α Cross-Talk in Hypoxia

Gut homeostasis involves an extensive functional cross-talk between HIF-1α and NF-κB. Presence of a putative NF-κB binding site was found on the HIF-1α promoter with potential role in transcriptional modulation and TNF-α induced NF-κB increases the HIF-1α mRNA basal level (vanUden et al., 2008). Furthermore, HIF-1α acts through negative feedback to NF-κB to reduce the host inflammatory response by restricting NF-κB dependent gene expression (Bandarra et al., 2015). Increase in NF-κB activity via HIF-1α depletion requires IKK and TAK1 (intermediately molecules in JAK and NF-κB signaling pathways, have kinase activity), whereby depleting HIF-1α in absence of TAK1 did not produce any depression of NF-κB activity. Association of TAK1with TAB activates IKK that leads to proteasomal degradation of IκBα (IκBα functions to inhibit the NF-κB) and results in NF-κB activation (Liu et al., 2017). Therefore, HIF-1α inhibits TAK1 to inhibit the downstream activation of IKK that in turn downregulates NF-κB activity for reduced inflammation (Figure 3). Hence, commensals could use this strategy for their survival and colonization inside the host.

#### Treg Differentiation

Regulatory T cells (Tregs) regulates immune system activation and act as a key component for the protection of the gut commensals against host immune response. Treg differentiation is of utmost crucial for the establishment and proliferation of the human gut microbiome. HIF-1α induces FoxP3 transcription that promotes the differentiation of naïve CD4^+^ cells into regulatory T-cells (Treg cells) (Luu et al., 2017) under cellular hypoxia (Hsu and Lai, 2018). Treg cells are well-known for their anti-inflammatory effects; they produce IL-10 (an anti-inflammatory cytokine) to downregulate the immune response (Lei et al., 2015). In other words, HIF-1α induces Treg differentiation that in turn reduces colonic inflammation and promotes immune tolerance fora hassle-free stay of gut residents (Luu et al., 2017) (Figure 3).

#### Impact of ATP Metabolites: Immunomodulation and Gut Barrier Regulation

Normally, ATP is located intracellularly, while during specific stress conditions or infections it is released extracellularly. Extracellular ATP functions as a signaling molecule by binding P2 purinergic cell surface receptors to provoke a pro-inflammatory immune response (Cekic and Linden, 2016). Extracellular ATP is further hydrolyzed to AMP by Nucleoside Triphosphate dephosphorylase or NTPDase (CD39) and finally to adenosine by Ecto-5'- Nucleotidase or 5-NT (CD73) to oppose the effects mediated by P2 receptors (Allard et al., 2017). Analysis of the CD73 gene promoter revealed the presence of HIF-1α binding site and its upregulation by hypoxia (Synnestvedt et al., 2002). Adenosine has an immune-modulatory role and improves epithelial barrier function by A2B adenosine receptor-induced phosphorylation of vasodilator-stimulated phosphoprotein (VASP). VASP was found protective in dextran sulfate sodium (DSS)-induced colitis (Aherne et al., 2015). Adenosine A3 receptor activation was found to attenuate TNF-α induced NF-κB signaling, as well as to improve the epithelial barrier functioning. Improved barrier function was reflected as an increase in TEER and expression of tight junction protein ZO-1(Ren and Zhou, 2017). Adenosine promotes a tolerogenic phenotype in macrophages. This tolerogenic phenotype is characterized by low expression of pro-inflammatory cytokines such as IL-12 and TNF-α while an increase in anti-inflammatory cytokines (IL-10) (Cekic and Linden, 2016). Adenosine also inhibits the LPS-induced production of pro-inflammatory cytokines by macrophages (Köröskényi et al., 2016). Adenosine thus complements the role of HIF-1α as a curator of gut homeostasis.

### HIF-1α and Inflammatory Gut Diseases

Integrity of the intestinal gut barrier is important for healthy living with significant role in quality of life. Inflammatory bowel disease (IBD) and celiac disease (CD) are prominent disease conditions impacting global population which involve the breakdown of the intestinal barrier with an unregulated inflammatory immune response. The leaky intestinal barrier allows exposure of microbial associated molecular patterns (MAMPs) to the Toll-Like Receptors to activate the host immune response (Jones and Neish, 2017). The host innate immune system facilitates superoxides generation (Kuwano et al., 2008). The generation of superoxides has been found to inhibit PHDs activity and promote the stabilization of HIF-1α (Hagen, 2012). The upregulation of HIF-1α has been functionally linked in both IBD and CD with significant role (Vannay et al., 2010). Stabilized HIF-1α exerts protective effects by downregulating the NF-κB signaling pathway to reduce cellular inflammation (Bandarra et al., 2015). HIF-1α has also been found upregulated in *Clostridium difficile* toxin exposed gut epithelial cells under normoxic conditions, as well as deletion of HIF-1α accounts for higher susceptibility toward *C. difficile* toxin-induced cellular damage (Hirota et al., 2010).

## Conclusion and Future Perspectives

**Figure 4:**
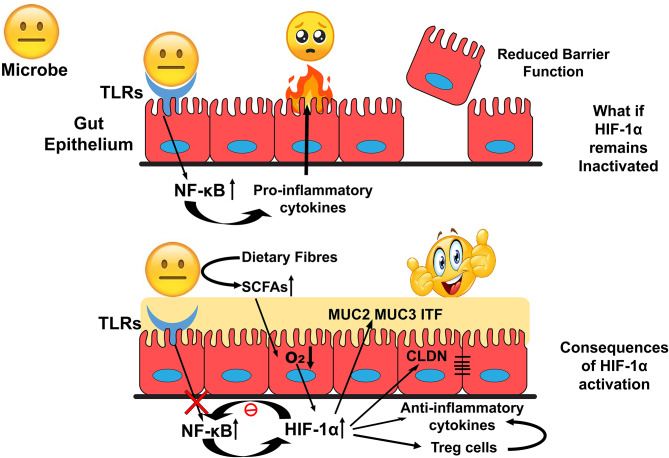
Overview of HIF-1α role in the maintenance of Gut Homeostasis. Inactivated HIF-1α could deteriorate intestinal epithelial integrity that exposes human commensals toward allows host immune response and develop an environment unsuitable for the colonization of the commensals. In contrast, activation of HIF-1α could modulate cellular bioprocesses to establish a favorable environment for their colonization.

HIF-1α upregulates genes involved in gut barrier functions such as *muc2, ITF, cldn1*, and other tight junction proteins. It is found to down-regulate the Nf-κB signalling and the production of anti-inflammatory cytokines to exert an immune-suppressive effect. These functions could favor the survival of gut residents and promote the interactions at the host-microbe interface (Figure 4). HIF-1α regulates several critical cellular processes and is a well-known therapeutic target for many human ailments. However, the role of HIF-1α in gut homeostasis has been recently elucidated and could be considered as a therapeutic target in microbial dysbiosis related human disorders. Further research toward this will bridge the gap between HIF-1α expression with the onset and prevention of inflammatory gut disorders in disease models.

## Author Contributions

NC and RP has conceived the idea, formulated the manuscript structure, and edited it. TK gathered the information and wrote the manuscript.

## Conflict of Interest

The authors declare that the research was conducted in the absence of any commercial or financial relationships that could be construed as a potential conflict of interest.
